# Impact of Grafting, Salinity and Irrigation Water Composition on Eggplant Fruit Yield and Ion Relations

**DOI:** 10.1038/s41598-019-55841-0

**Published:** 2019-12-18

**Authors:** Gülüzar Duygu Semiz, Donald L. Suarez

**Affiliations:** 1 Ankara University, Agricultural Faculty, Dept. of Agricultural Structures and Irrigation, Ankara, Turkey; 2 USDA-ARS Salinity Laboratory 450 W, Big Springs Road, Riverside, CA 92507 USA

**Keywords:** Abiotic, Salt

## Abstract

Scarcity of fresh water in arid and semi-arid regions means that we must use more saline waters for irrigation and develop tools to improve crop salt tolerance. The objectives of our study were to (1) Evaluate fruit production, salt tolerance and ion composition of eggplant cv Angela, both nongrafted and when grafted on tomato cv Maxifort rootstock and (2) Evaluate eggplant specific toxicity effect of Cl^−^ and Na^+^ ions under saline conditions. We salinized the irrigation water with either a Na^+^-Ca^2+^- Cl^−^ composition typical of coastal Mediterranean ground waters as well as a mixed Na^+^-Ca^2+^-SO_4_^2−^ Cl^−^ type water, a composition more typical of interior continental basin ground. For each water type we evaluated 5 different salinity (osmotic) levels of –0.003 (control), –0.15, –0.30, –0.45 and –0.60 MPa. There were no statistically significant differences in the fruit yield relative to the water type, indicating that Cl^−^ ion toxicity is not a major factor in eggplant yield associated with salinity. This conclusion was confirmed by the determination that leaf Cl content was not correlated with relative yield. The electrical conductivity of the saturation extract (EC_e_) at which yield is predicted to be reduced by 50% was 4.6 dS m^−1^ for the grafted plants vs. 1.33 dS m^−1^ for the nongrafted plants. The relative yield was very well correlated to leaf Na concentrations regardless of grafting status, indicating that Na is the toxic ion responsible for eggplant yield loss under saline conditions. The increased salt tolerance of cv Angela eggplant when grafted onto tomato Maxifort rootstock is attributed to a reduced Na uptake and increased Ca and K uptake with Maxifort rootstock.

## Introduction

Semiarid regions of the world have a scarcity of good-quality water. Competitive demand for fresh water among urban, industrial, and agricultural sectors has led researchers to focus on using marginal waters for agricultural production^1^. There have been many research efforts to improve the salt tolerance of crops by traditional breeding programs. However, commercial success has been very limited as salt tolerance is complex genetically and physiologically^2,3^. Factors limiting plant growth under saline conditions may vary among crops, requiring crop specific information before traditional breeding or marker assisted breeding can be successful. Furthermore, lack of general public acceptance of genetic engineering for crop improvement means that other approaches to improving yield under saline conditions need to be considered^3^.

Increased tolerance to salinity was related to reduced Na^+^ in shoot tissues with no change in Cl^−^ ^4^ reduced concentrations of Na^+^ and Cl^−^ ^3^, and increased K^+^ with lower Na^+^ and Cl^−^ ^5^. Surprisingly a large percentage of salinity studies on horticultural or agronomic crops use NaCl as the sole salinizing agent. The use of these single salt salinizing compositions may limit the extent to which the results can transferred to field conditions, especially if the salinity damage is caused by specific toxic ions rather than osmotic effects. The same argument can be made for the anion as well as cation composition^6^. Munns and Testor^7^ considered that several processes were involved with salt tolerance, primarily the effects of osmotic stress and secondarily tissue ion tolerance.

Most published salinity and grafting experiments were conducted solely with NaCl as the salinizing salt^3,8,9^. Penella *et al*.^10^ conducted a field experiment with grafted and nongrafted pepper using a mostly NaCl irrigation water at EC = 7.5 dS m^−1^. The salt tolerance of eggplant has been investigated earlier, primarily with NaCl as the salinizing solution. Assaha *et al*.^11^ measured a 49% decrease in vegetative growth when eggplant was irrigated for 14 d with 50 mM NaCl, Heuer *et al*.^12^ in a field experiment determined that fruit yield decreased above EC_e_ = 1.1 dS m^−1^. Based on his salinity response equation we calculate that he had a 50% yield loss at EC_e_ = 8.3 dS m^−1^. Hannachi *et al*.^13^ measured differences in salt tolerance among four eggplant cultivars. Akinci *et al*.^14^ determined that NaCl salinity decreased germination and seedling dry weight of three eggplant cultivars while leaf Na increased (leaf Cl not determined). We found no studies that investigated specific anion effects in relation to salinity tolerance of grafted eggplant in terms of yield. Earlier studies on tomato have found no significant effects comparing self-grafted and nongrafted rootstock of tomato indicating that grafting per se had no impact on fruit yield^9,15^, or qualitative fruit parameters and measured fruit ion composition^15^. These findings are consistent with the results of Fernandez-Garcia *et al*.^16^ that xylem and phloem vessels are formed through the graft union 8 d after grafting. Under saline conditions there were no differences in tomato fruit yield between nongrafted and self grafted plants^3,9^, indicating that salt tolerance is also unaffected by grafting per se.

The objective of our study was to (1) Evaluate the yield and salt tolerance of eggplant, cv Angela, under both nongrafted conditions and when grafted on tomato cv Maxifort rootstock and (2) Evaluate eggplant specific toxicity effect of Cl^−^ and Na^+^ ions and K/Na relatons under saline conditions. The effects of salt ion composition on plant salt tolerance are important for improved prediction of crop response to salinity under field conditions and for identifying specific ion toxicity for future salt tolerance breeding.

## Material and Methods

The study was carried out in a greenhouse within 30 sand tanks (1.2 length × 0.6 width × 0.5 m deep) in USDA Salinity Laboratory., Riverside, California. Two grafted and two nongrafted seedlings were sown in each sand tank. The sand tanks were flood irrigated with the same amount of water (approximately 130 L) once a day. The daily flushing of the sand means that the rootzone salinity was maintained essentially equal to the irrigation water (reservoir) salinity. Drainage water returned to the same reservoir (1,500 L) after each irrigation. Irrigation water quality checks performed daily (EC and pH) and weekly sampled and analyzed to monitor ion content. Addition of deionized water was generally sufficient to maintain ion composition (with minor nitric acid and KCl addition to maintain pH, nitrate and K concentrations. Micronutrients were also reapplied approximately midway through the experiment. Salinity treatments were initiated two weeks after planting. Grafted and nongrafted eggplant seedling were purchased from Bevo Farms (Milner, BC, Canada). Eggplant seedlings consisted of nongrafted cv Angela and grafted cv Angela scions onto cv Maxifort tomato rootstock. Salinity treatments were undertaken at osmotic potential (OP) levels of –0.003 (control), –0.15, –0.30, –0.45 and –0.60 MPa.

In order to examine the effects of different source of salts at the same OP level, we prepared SO_4_^2−^Cl^−^ and Cl^−^ irrigation waters to represent two water types. The salt treatments were prepared for the two water types, either equal (in mmol_c_ L^−1^) concentrations of Ca^2+^ and Na^+^ with Cl^−^ as the anion, designated as a Cl^−^ water, or a mixed salt solution better representing arid zone of the world, designated as a SO_4_^2−^Cl^−^ water. The SO_4_^2−^Cl^−^ water had a relatively high SO_4_^2−^ concentration, Na^+^ > Ca^2+^ at high salinity, and increasing Mg^2+^ with increasing salinity^17^. The calculations to obtain equal osmotic values for the two water types were made using the Extract Chem model^18^. The composition of irrigation waters is shown in Table 1. Modified half Hoagland’s solution (plant nutrient solution) included 0.17 mmol L^−1^ KH_2_PO_4_, 0.75 mmol L^−1^ MgSO_4_∙7H_2_O, 2.0 mmol L^−1^ KNO_3_, and 0.25 mmol L^−1^ CaSO_4_. 2H_2_O with micronutrients, also expressed in mmol L^−1^, of 0.34 KH_2_PO_4_, 0.050 Fe (as sodium ferric diethylenetriamine pentaacetate), 0.023 H_3_BO_3_, 0.005 MnSO_4_, 0.0004 ZnSO_4_, 0.0002 CuSO_4_, and 0.0001 H_2_MoO_4_.

**Table 1:** Irrigation water compositions.

OP (MPa)	dSm^−1^		mmol_c_ L^−1^							
	EC	pH	Na^+^	K^+^	Ca^2+^	Mg^2+^	SO_4_^2−^	Cl^−^	PO_4_^3−^	NO_3_^−^
Control (−0.03)	1.20	4.90	1.5	3.0	4.0	2.0	2.0	3.0	1.5	5
−0.15 Cl^−^	4.00	4.92	16	3.0	16	2.0	2.0	29	1.5	5
−0.30Cl^−^	8.26	4.92	36	3.0	36	2.0	2.0	69	1.5	5
−0.45 Cl^−^	12.0	4.93	55.5	3.0	55.5	2.0	2.0	109	1.5	5
−0.60Cl^−^	15.8	4.94	75	3.0	75	2.0	2.0	148	1.5	5
Control (−0.03)	1.20	4.90	1.5	3.0	4	2.0	2.0	3.0	1.5	5
−0.15 SO_4_^2−^ Cl^−^	4.33	4.93	15.7	3.0	14.7	7.6	19.8	20.8	1.5	5
−0.30 SO_4_^−2^ Cl^−^	8.88	4.93	32	3.0	32	16	45.5	45.5	1.5	5
−0.45 SO_4_^−2^ Cl^−^	12.2	4.92	51	3	35	26	56.9	75.	1.5	5
−0.60 SO_4_^−2^ Cl^−^	15.8	4.93	74	3.0	36	33	65.4	105.5	1.5	5

Irrigation water composition including Na, K, Ca, Mg, S, Fe, Mn, Cu, and Zn determined using PerkinElmer Optima 3300DV ICP OES (inductively coupled plasma optical emission spectroscopy) (PerkinElmer Corp, Waltham, MA, USA), and Cl by amperometric titration using a Labconco chloridometer (Labconco, Kansas City MO, USA) and NO^3−^ by spectrophotometrically using a Hitachi model 100-20 (Hitachi Corp, Japan) at a 210 nm wavelength. The plant and fruit samples were washed in deionized water, dried in a forced-air oven at 70 °C for 72 h, and ground in a Wiley mill to pass a 60 mesh screen. Total S, total P, Ca, Mg, Na, and K of the leaf and fruit tissue were determined from nitric–perchloric acid digests of the tissues by ICP OES. The fruit and leaf Cl^−^ was determined on nitric–acetic acid extracts by amperometric titration. Statistical analyses of all data were performed using SPSS 16.0 (SPSS Inc., Chicago, IL, USA). The salt tolerance models suggested by Maas and Hoffman^19^ and van Genuchten and Hoffman^20^ were used to evaluate the salinity tolerance for grafted and nongrafted plant respectively.

## Results

### Yield

The yield data, presented in Table 2 show higher fruit yields for Maxifort grafted plants relative to nongrafted plants at all osmotic pressures. The statistical results on the yield data indicate that there were no significant differences in yield based on water type (p < 0.05) thus subsequent analysis considered the entire data set.

**Table 2:** Fruit yield of grafted and nongrafted eggplant as related to osmotic pressure (salinity).

	OP, MPa Salinity, dSm^−1^	−0.03 1.1	−0.15 3.98	−0.30 8.26	−0.45 12.02	−0.60 15.84	Mean
Yield, kg plant ^−1^	Salinity	9.89 A	6.21 B	3.45 C	2.64 C	1.55 C	
	Grafted	11.16 a A	10.39 a A	5.21 a B	3.74 a BC	2.23 a C	6.546 a
	Nongrafted	8.62 b A	2.03 b B	1.66 b B	1.54 a B	0.86 a B	2.94 b

A → , a↓, Grafting, salinity, grafting x salinity, salt x salinity, significance, p < 0.005.

Grafting, salinity and interactions of grafting x salinity had significant effects on yield at p < 0.05 level (Table 2). The highest yield was obtained from the grafted control treatment with 11.16 kg plant^−1^. In our experiment, Maxifort grafted plants had yield decreases of 6.8, 53, 66 and 80%, relative to the control, for salinity levels of −0.15, −0.30, −0.45 and −0.60 MPa, respectively. The significant interaction of grafting x salinity is evidenced by the data that grafted plants had slightly higher yields under control and much higher yields under OP levels of −0.15 to −0.45 MPa (corresponding to EC values of 4 to 12 dS m^−1^). For the control treatment in the absence of salt stress, the yield difference was 22.8% greater for the Maxifort grafted plants as compared to nongrafted plants. The yield differences between Maxifort grafted and nongrafted plants were much more prominent under moderate and high salinity, with the yield differences at 3.98 and 8.26 dSm^−1^ being 80.5 and 68.1%, respectively. Thus, in our experiment, the positive effect induced by grafting tomato (cv Maxifort) rootstock on eggplant fruit salt tolerance increased with the level of salt stress.

### Ion uptake in leaves and fruits

Statistical examination revealed that there was a significant (p < 0.001) difference in Na^+^ shoot content between Maxifort grafted and nongrafted plants, with grafted plants having lower Na^+^ uptake (Table 3). There were no statistical differences in Na^+^ content in leaves related to water type (data not shown) so the data were combined. This is not unexpected as the two water compositions have similar Na^+^ ion compositions (Table 1). In the control treatment sodium uptake for grafted and nongrafted plants was not statistically different (Table 3). Increasing irrigation water salinity led to an increase in Na^+^ uptake by both grafted and nongrafted plants, and at any salinity level Na^+^ uptake in leaves was significantly and much lower for grafted plants (Table 3). Similarly, fruit Na^+^ contents were not significantly different for water type but were significantly greater in nongrafted as compared to grafted plants (Table 4). Except for the control treatment, Cl^−^ content in leaves were higher for treatments irrigated with Cl^−^ as compared to SO_4_^2—^Cl^−^ waters, again consistent with the water compositions. Calcium accumulation in leaves was affected by both water type and grafting (Table 3). The Cl^−^ salt treatment had slightly (12.4%) but significantly greater Ca^2+^ on average as compared to the SO_4_^2+^ – Cl^−^ treatment (Table 3), consistent with the greater Ca^2+^ in the Cl^−^ salt irrigation waters. The average leaf Ca^2+^ content of grafted eggplants was much greater (40%) and highly significantly different than nongrafted plants, p < 0.001 (Table 3). Statistical analyses revealed that the mean Ca^2+^ content of the fruit was greater for grafted as compared to nongrafted plants (Table 4). The fruit Ca^2+^ content decreased with salinity (Table 4).

**Table 3:** Ion composition of leaves as related to osmotic pressure (salinity) and grafting.

	Osmotic Potential (MPa)	−0.003	−0.15	−0.30	−0.45	−0.60	Average
Leaf Na, mmol kg^−1^	Grafted	21.1 D a	52.1 C b	67.8 C b	90.4 B b	145 A b	75.2 b
	Nongrafted	22.8 D a	76.1 CD a	155 BC a	192 B a	364 A a	162.0 a
	Average (Salinity)	22.0 D	64 CD	111.BC	141 B	254 A	
	↓a, → A, salinity p < 0.001, grafting p < 0.001, Interaction (Grafting*Salinity) p < 0.001,						
Leaf Cl, mmol kg^−1^	Grafted	299 D b	420.C b	601 B b	824 AB b	1360 A b	700.5 b
	Nongrafted	598 D a	1050 CD a	1160 BC a	1449 B a	1750 A a	1202.400 a
	Cl^−^	561 D **a**	849 C **a**	1090B **a**	1523 A **a**	1630A **a**	10853 **a**
	SO_4_	336 C **a**	622 B **a**	678 B **b**	750 B **b**	1480 A **a**	818. **b**
	Salinity (Ave)	448 D	735 C	882 BC	1140 B	156 A	
	↓a, ↓ **a**, → A, salinity p < 0.001, grafting p < 0.001, Interaction (Grafting*Salinity) p < 0.001,						
Leaf Ca, mmol kg^−1^	Cl	1540 A a	1640 A a	1790 A a	1720 A a	1740 A a	1687.13 a
	SO_4_	1670 A a	1524 A b	1405 A a	1370 B b	1420 A a	1477.17 b
	Grafted	2000	2000	2010	1880	1960	1967.33 **a**
	Nongrafted	1220	1170	1190	1210	12110	1200.54 **b**
	↓a, ↓ **a**, → A, grafting p < 0.001, Salt p < 0.001, Interaction (Salt*Salinity) p < 0.005,						
Leaf Mg, mmol kg^−1^	Cl	311 A a	281 A b	231 A b	168 B b	265 A b	251.27 b
	SO_4_	279 C a	378 B a	385 B a	514 A a	487 A a	408.57 a
	Grafted	387 A a	438 A a	394 A a	423 A a	420 A a	412.30 a
	Nongrafted	204B b	222 B b	221 B b	259 B b	332 A b	247.53 b
	Average (Salinity)	295 B	330 AB	308 B	341 A	376 A	
	↓a, → A, salinity p < 0.001, grafting p < 0.001, salt p < 0.001, Interaction (Grafting*Salinity) p < 0.001, (Salt*Salinity) p < 0.001						
Leaf, P, mmol kg^−1^	Grafted	145 A a	99.5 A a	81.7 A a	116 A a	107 A b	
	Nongrafted	107 B a	132. AB a	106 B a	100 B a	183 A a	
	↓a, → A, Interaction (Grafting*Salinity) p < 0.05						
Leaf, K, mmol kg^−1^	Grafted	1909	1745	1541	1566	1405	1633.13 a
	Nonngrafted	1000	973	957	1031	1048	1001.63 b
	↓a, grafting p < 0.001						
Leaf, S, mmol kg^−1^	Grafted-Cl	157 b A *a*	102 b A *a*	92.7 b AB *a*	85.6 b B *a*	124 b AB *a*	
	Grafted-SO4	229 a B a	308 a AB a	343 a A a	318 a AB a	332 a A a	
	Nongrafted-Cl	64.0 **a** A *b*	67.9 **a** A *a*	78.7 **a** A *a*	70.8 **a** A *a*	92.5 **a** A *a*	
	Nongrafted-SO_4_	63.0**a** A b	69.6 **a**A b	75.9 **a** A b	93.4 **a** A b	92.3 **a** A b	
	Salinity (ave)	128.2 B	137.1 A	147.5 A	142.0 A	160.2 A	
	↓**a**, ↓*a*, ↓a, ↓a, → A, salt p < 0.001, salinity p < 0.05, grafting p < 0.001, Interaction (salt*grafting) p < 0.001, (Salt*Salinity) p < 0.001 (Salt*Salinity*grafting) p < 0.001

**Table 4:** Ion composition of eggplant fruit as related to salinity and grafting.

Na, mmol kg^−1^	Grafted	21.5 C a	29.0 BC a	34.7 AB b	40.2 AB b	48.5 A b	34.79 b	
	Nongrafted	21.3 C a	33.9 C a	52.1 B a	51.6 B a	77.0 A a	47.18 a	
	Average(salinity)	21.45 D	31.42 C	43.40 B	45.88 B	62.77 A		
	↓a, → A, salt p < 0.05, salinity p < 0.001, grafting p < 0.001, Interaction (Grafting*Salinity) p < 0.001							
Cl, mmol kg^−1^	Grafted	160	148	166	199	200	219.27 **b**	
	Nongrafted	124	210	239	235	253	167.40 **a**	
	Cl	163 a C	203 a BA	226 a A	248 a A	235 a A	206.63 a	
	SO4	122 b B	155 b A	179 b A	186 b A	218 a A	180.03 b	
	Salinity	142 B	179 AB	202 A	217 A	226 A		
	↓a, ↓ **a**, → A, salt p < 0.001, salinity p < 0.001, grafting p < 0.001, Interaction (Salt*Salinity) p < 0.001							
Ca, mmol kg^−1^	Grafted	68.2	62.5	44.5	30.2	29.5	46.99 a	
	Nongrafted	55.0	49.6	37.8	32.3	27.6	40.46 b	
	Average (salinity)	61.6 A	56.0 B	41.2 CD	31.3 DE	28.6 E		
	↓a, → A, salinity p < 0.001, grafting p < 0.01							
Mg, mmol kg^−1^	Cl	92.8 A a	87.2 A a	79.8 A a	79.1 A b	74.2 A b	82.7 b	
	SO_4_	92.2 A a	86.5 A a	89.4 A a	93.3 A a	98.1 A a	92.0 a	
	↓a, → A, salt p < 0.01, Interaction (Salt*Salinity) p < 0.05							
P, mmol kg^−1^	Cl	142 AB a	149 A a	124 B a	130 AB a	132 AB a	135.44 a	
	SO_4_	135 A a	112 B b	125 AB a	126 AB a	137 A a	127.25 b	
	↓a, → A, salt p < 0.05, grafting p < 0.001, Interaction (Salt*Salinity) p < 0.005							
K mmol kg^−1^	Cl	887 AB a	952 A a	839 AB a	860 AB a	807 B a		
	SO_4_	873 A a	816 A b	886 A a	863 A a	840 A a		
	↓a, → A, grafting p < 0.001, Interaction (Salt*Salinity) p < 0.05							
S, mmol kg^−1^	Grafted-Cl	68.0	64.7	60.3	61.0	63.8	59.24 b	
	Nongrafted-Cl	59.5	59.7	49.8	54.9	50.7		
	Grafted-SO_4_	72.5	73.0	70.5	62.9	72.0	63.46 a	
	Nongrafted-SO_4_	54.5	51.8	54.2	61.5	61.6		
	↓a, → A, salt p < 0.001, grafting p < 0.001							

Leaf Mg^2+^ contents were affected by salinity, water type and grafting. There were grafting x salinity and salt x salinity interactions (Table 3). The Mg^2+^ content for grafting was greater than for nongrafted plants (Table 3). Consistent with leaf Mg accumulation, average fruit Mg^2+^ concentration was also higher in the SO_4_^2−^Cl^−^ salt treatments (Table 4). Leaf and fruit S accumulation were also affected by water type, as SO_4_^2−^Cl^−^ water treatments had higher S in leaves and fruits as compared to the Cl^−^ irrigation water treatments (Tables 3 and 4).

Leaf K^+^ concentrations were significantly and much higher in the grafted plants as compared to the nongrafted plants (Table 3) for the controls as well as for all salinity treatments, however K^+^ was in all instances above deficiency levels, (<20–50 mg kg^−1^ vegetative dry weight, Marschner^21^) and there were no visual symptoms of K^+^ deficiency.

The P content in leaves was in general not significantly greater in nongrafted Angela plants as compared to Maxifort grafted plants across all salinity concentrations (Table 3) nor was there any indication of P deficiency. Fruit P concentrations were not significantly different as related to salinity or grafting, but were slightly greater on average in Cl as compared to SO_4_ type irrigation waters (Table 4).

Leaf Cl contents were affected by salinity, grafting and water type. Consistent with increased Cl content in the Cl type waters, these treatments had increased leaf Cl. Nongrafted plants had significantly greater leaf Cl concentrations and Cl content increased with salinity (Table 3). In contrast Cl content in fruit from nongrafted plants was significantly lower than Cl content in Maxifort grafted plants (Table 4).

## Discussion

The lack of a significant difference in fruit yield between the Cl^−^ and the SO_4_^2−^Cl^−^ irrigation water types means that Cl^−^ as a specific toxic ion was not an important factor affecting eggplant yield under saline conditions. The much greater Cl^−^ concentration in the Na^+^-Ca^2+^-Cl^−^ irrigation water did not cause a significant decrease in yield.

Earlier, Savvas and Lenz^22^ found no difference in eggplant yield between plants salinized with NaCl and those salinized with nutrient solution. They concluded that eggplant response to salinity was related to osmotic rather than toxic ion effects. However they examined only a control and one salinity level (EC = 4.7 dS m^−1^) and under the saline treatments (with or without added nutrients) they found only a 19% total fruit yield loss relative to the control. Thus the yield loss under salt stress in their experiment may not have been sufficiently large to detect differences in yield related to ion composition. In our experiment the yield loss of grafted plants at EC = 4.0 dS m^−1^ was also low, only 7% yield loss relative to control, and also not significantly different from the control (Table 2).

In our experiment, eggplant grafted to cv. Maxifort tomato rootstock had increased yield under control conditions relative to nongrafted eggplant, but the relative differences were much greater under saline conditions. The increased differences in eggplant yield that we observed with increasing stress, has been observed earlier in tomato when one genotype was grafted onto another more tolerant rootstock^3,9,23^. Compared with the control, the yield decreases for Maxifort grafted plants were 6.8, 53, 66.5, 80% while they were 76.5, 80, 82 and 90% for nongrafted plants, respectively (Table 2).

### Salinity tolerance

Salt tolerance can often be adequately described on the basis of two parameters: threshold, and slope^20^. Typically these data are expressed as EC_e_, the EC of a saturation extract as shown in Eq. 1

1 $$\frac{Y}{{Y}_{0}}=100-b(E{C}_{e}-a)$$

**Figure 1:**
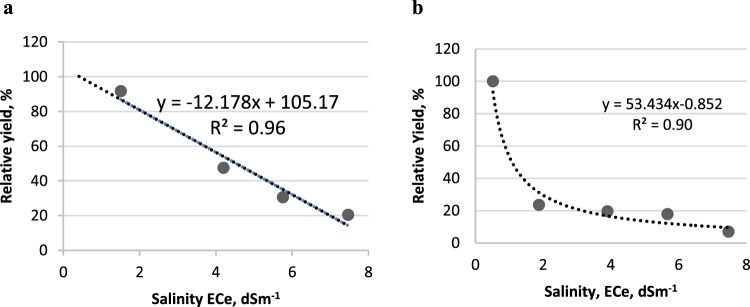
Relative yield and salt tolerance model for (**a)** Maxifort grafted on Angela eggplant (linear response, using Maas-Hoffman (1977) model) and (**b**) nongrafted eggplant (exponential response using van Genuchten -Hoffman (1984) model).

Where Y/Yo is relative yield, a is the threshold EC salinity level at which yield starts to decline, b is the slope of the response curve expressed as % yield loss per dS m^−1^ and EC_e_ is the EC of the saturation extract. In our experimental sand tank system the relation between EC_iw_ and EC_e_ (saturated paste) was calculated as EC_e_ = 0.472 EC_iw_^24^. As seen in Fig. 1a, the Maas-Hoffman model well represented the salinity response for grafted plants (R^2^ = 0.96). The model provides a salinity threshold value of 0.42 dS m^−1^ with a 12.2% slope. The predicted EC_e_ for 50% yield is 4.6 dS m^−1^. The Maas-Hoffman model was not satisfactory for representing the salinity response of nongrafted plants, primarily because the yield loss was very extensive between the control and the first salinity treatment and the data were highly non-linear. In this instance we analyzed the response using the van Genuchten and Hoffman^20^ model, shown below

2 $${\alpha }_{s}=\frac{1}{1+{(\frac{h}{{h}_{50}})}^{b}}$$

where α_s_ is the dimensionless stress response function (relative yield), *h* is the osmotic stress and *h*_50_ is the stress value at which there is a 50% yield loss, and *b* is an empirical fitting parameter. Using this model, expressed in terms of EC_e_ rather than OP, we optimized the EC_50_ to the observed biomass data at the different salinities using TableCurve 2D version 5.0^25^. The 50% yield loss corresponded to EC_e_ = 1.33 dSm^−1^ (Fig. 1b) and the model fit resulted in a good prediction (R^2^ = 0.90). The yield data clearly showed that the Maxifort grafted plants had significantly greater yield than nongrafted plants (Table 2) and the salt tolerance analysis showed that salt tolerance was also greater for grafted plants (Fig. 1). The increased salt tolerance in Maxifort grafted plants is evidenced by the calculated 50% yield loss at 4.6 dS m^−1^ as compared to a 50% yield loss at 1.33 dS m^−1^ in the nongrafted Angela eggplant.

**Figure 2:**
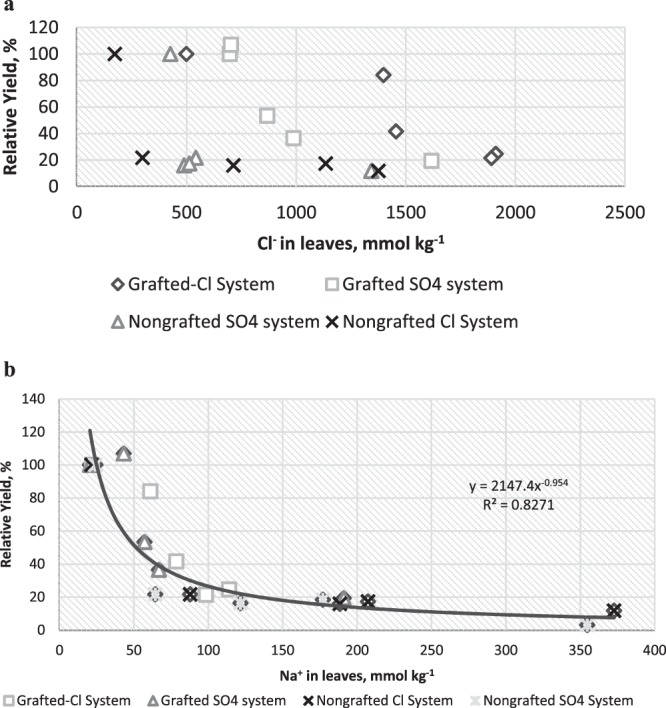
Eggplant fruit yield as related to, (**a)** Cl^−^ ion content and (**b**) Na^+^ ion content for eggplant grafted onto Maxifort rootstock, nongrafted and irrigated with Cl^−^ or SO_4_^2−^Cl^−^ type water.

In a short term experiment Assaha *et al*.^11^ measured a 49% decrease in leaf dry weight for eggplant irrigated with 50 mM NaCl solutions relative to the non-saline control. This value corresponds to an approximate EC_e_ of 2.5 dS m^−1^, but direct comparison with our data was not possible as they measured vegetative growth not fruit yield. In a field experiment, the relative yield of eggplant cv Black Oval, was reported as 72% of control at an EC_e_ = 4.7 dS m^−1^ ^12^ thus somewhat more salt tolerant than even our grafted plants. These comparisons suggest that either varietal differences in eggplant salt tolerance are quite large, and/or that under greenhouse conditions in our study, there was less stress from other abiotic factors thus salt stress was more evident than under field conditions. Since earlier research established that grafting per se does not affect yield and water and ion uptake, we attribute the effect of grafting to increased salt tolerance of Maxifort tomato as compared to Angela eggplant. Tomato appears to be more salt tolerant than eggplant. For example under experimental conditions similar to this study, Big Dena tomato had a 50% fruit yield loss at EC_e_ = 6.6 dS m^−1^ ^17^ as compared to EC_e_ = 1.33 for Angela eggplant (Fig. 2). Grafting Maxifort rootstock onto Big Dena tomato resulted in 50% tomato yield loss at EC_e_ 5.7 dS m^−1^ ^17^ a value close to that found for eggplant grafted to Maxifort rootstock in this study (EC_e_ = 4.6 dS m^−1^). Thus the salt tolerance response of Maxifort grated eggplant is similar to the response of Maxifort grafted on another tomato variety.

### Ion uptake in leaves and fruits

As there is little data on the relation of tissue ion composition and salt tolerance for eggplant we primarily must relate our data to findings reported for other solanaceae species (tomato and pepper). Low accumulation of Na^+^ and/or Cl^−^ in the shoot is frequently a characteristic of salt tolerant grafted tomato plants^3,4,17,26^. However Al-Harbi *et al*.^27^ found no decrease in Na^+^ or Cl^−^ contents on grafted tomato and Penella *et al*.^10^ found that grafting a salt tolerant pepper rootstock on a commercial pepper variety increased yield and photosynthesis parameters but did not decrease Na^+^ and Cl^-^ leaf ion content as compared to nongrafted pepper. Penella *et al*.^28^ determined increased salt tolerance of grafted pepper as compared to nongrafted pepper and significantly greater Cl^−^ but not Na^+^ in the leaves of nongrafted plants. Although these experiments did not evaluate different irrigation water compositions, it seems most likely that the specific response to grafting pepper and tomato to ion uptake depends on the specific characteristics of the rootstock relative to the scion and might not be generalized, as commented earlier by others^29^.

The shoot ion content results from our experiment, where nongrafted plants had significantly higher Na^+^ concentrations are consistent with Na^+^ ion toxicity as yield and salt tolerance differences were greater for the grafted plants. Trends in fruit Na^+^ content in our experiment were also consistent with the leaf Na^+^ content. Grafting with Maxifort rootstock restricted Na^+^ accumulation in eggplant fruits. The differences in Na uptake between grafted and nongrafted plants may thus be an explanation of the increased salt tolerance of Maxifort grafted eggplants. This result is also consistent with the finding that cv Big Dena tomato scion grafted onto cv Maxifort tomato rootstock tends to exclude Na^+^ ions relative to cv Big Dena tomato^15^. Also, Bai *et al*.^30^ reported that eggplant grafted onto S. torvum had lower leaf Na^+^ and Cl^−^ contents than did self-rooted plants under NaCl stress. These results are in contrast to Almeida *et al*.^31^ who in a three week experiment did not find a correlation between Na^+^ concentrations in the leaves and vegetative growth of 23 tomato accessions. They concluded that the relationship between Na^+^ concentration in the cells and tissue tolerance to salinity may vary among accessions. Our results are also in contrast to those of Huertas *et al*.^32^ who determined that in a five day experiment transgenic tomato with overexpression of SISOS2 gene was associated with *higher* Na^+^ content in leaves and higher growth relative to control. However Rivero *et al*.^33^ determined that tomato plants maintained higher K^+^/Na^+^ ratios, and reduced Na^+^ as well as better physiological response in combined heat-salinity stress treatments as compared to salinity only treatments. Their four day experiment on 30 d old plants could not evaluate the long term effects on biomass and yield. However, they did measure increased CO_2_ assimilation and increased transpiration in the combined stress treatments, consistent with greater K^+^/Na^+^ ratios less adverse salinity effects. Assaha *et al*.^11^ compared vegetative growth and ion uptake of eggplant and huckleberry under two levels of NaCl. The smaller decrease in relative growth of huckleberry as compared to eggplant was attributed to the lower leaf Na^+^ concentration in huckleberry however they did not evaluate leaf Cl^−^ concentrations.

Leaf Cl^−^ content in our experiment increased with increasing salinity but Maxifort grafted plants had lower leaf Cl^−^ as compared to nongrafted plants at all salinity levels. As found for Na^+^, the Cl^−^ levels were approximately double in the nongrafted as compared to grafted plants. On this basis alone we cannot distinguish Na^+^ from Cl^−^ ion toxicity. These results are thus similar to those found by Estan *et al*.^3^ for grafted tomato and Bai *et al*.^30^ for eggplant. Penella *et al*.^28^ observed that grafted plants (irrigated with 40 mM NaCl) that were more salt tolerant than other grafted combinations of pepper, had lower Na and Cl content in the scion. They attributed the improved salt tolerance on ability to restrict toxic ions Na^+^ and Cl^−^ ions from either entering the roots or being transported to the leaves rather than through synthesis of osmotically active metabolites^28^. Other researchers have also determined that more salt tolerant cultivars of rootstocks of grafted plants had reduced leaf Na and Cl content for pepper^13^, for tomato^9,34^ reduced Na for tomato (Cl not analyzed)^35^, reduced Na but not reduced Cl for tomato^17^. With the exception of Semiz and Suarez^17^, earlier studies on tomato, eggplant and pepper used a single salt as the salinizing solution (almost always NaCl), thus they could not evaluate relative toxicity of Na^+^ and Cl^−^ ions.

In our experiment we can evaluate the relative importance of Na^+^ vs Cl^−^ ions by examination of the results of irrigation with two water types. The SO_4_^2−^Cl^−^ water type had yields that were not statistically different across salinity levels from the Cl^−^ type waters. Thus the Maxifort grafted plants had greater salt tolerance than nongrafted Angela eggplant under both water types, providing strong evidence that Na is the growth limiting toxic ion under these salinity levels. Furthermore, analysis of the relationship between leaf Na^+^ and relative yield and leaf Cl^−^ and relative yield, evaluating different salinities, grafting and water types, shows that leaf Cl^−^ was not a predictor of eggplant yield (Fig. 2a) This statistical analysis is consistent with the determination that the Cl^−^ salt treatment waters did not have significantly different yield than the SO_4_^2−^Cl^−^ salt treatment waters. We conclude that Cl^−^ ion toxicity is not the major source of yield loss in eggplant.

In contrast to the lack of a relationship between leaf Cl^−^ and relative yield, there was a good relationship between leaf Na^+^ and relative yield, representing all four treatments. The yields, and salt tolerance of these treatments were very different yet as shown in Fig. 2b (R^2^ = 0.83), relative yield was well predicted by leaf Na^+^ concentration (across water types, salinity and grafting). These data strongly indicate that Na^+^ ion is the yield limiting toxic ion in eggplant and the Maxifort grafted plants increased salt tolerance relative to nongrafted Angela can be attributed to improved Na^+^ exclusion of the Maxifort rootstock.

**Figure 3:**
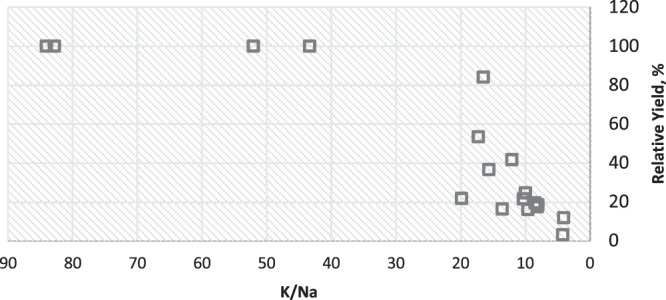
Eggplant fruit yield as related to leaf K/Na ratio for Maxifort grafted and nongrafted Angela eggplant irrigated with Cl^−^ or SO_4_^2−^Cl^−^ type water.

Many studies have indicated that maintenance of a high K/Na ratio is critical to salt tolerance, uptake^9,13,35,36^. Li *et al*.^37^ examined a salt tolerant and salt sensitive eggplant cultivar under 200 mM NaCl. They observed that the salt tolerant cultivar had a higher K/Na ratio in the leaves as well as upregulation of seven genes related to K^+^ transport. They concluded that K uptake is more important than Na exclusion because only one Na transporter was regulated in a similar fashion. However, rather than counting number of genes upregulated, if we examine the ion content in there study we find approximately equal changes in Na and K. We calculate that at the end of their experiment (23 d of exposure to salinity), the salt tolerant cultivar had 50% of the Na content and 180% of the K content of the salt sensitive cultivar. In our study grafted plants had 47% and 40% of the Na content of the nongrafted plants at the two highest salinity levels, respectively and 152 and 130% of the K leaf content of nongrafted plants respectively. Thus greater K content as well as lower Na content in eggplant is associated with increased salt tolerance. The relationship between K/Na vs. fruit yield is shown in Fig. 3 for all treatments. There is a relationship of decreasing yield below K/Na ratios of 22 but the relationship is not as good as that between Na and yield (Fig. 2b). The increase in leaf K was much greater than the decrease in leaf Na at each salinity level (Table 2) so it is not simply a case of improved K/Na selectivity. Our results are consistent with the findings of others that salt tolerant varieties or salt tolerant grafted plants had increased K/Na selectivity. The grafting of Maxifort rootstock on Angela thus results in substitution of K^+^ for Na^+^ ions, which is beneficial to adaption to saline conditions, minimizing Na^+^ toxicity while maintaining osmotic adjustment.

The increased Ca^2+^ uptake in grafted plants was associated with a corresponding decrease in Na^+^ uptake, suggesting that the mechanism reducing Na^+^ uptake in grafted plants was also related to Ca^2+^/Na^+^ selectivity. The greater uptake of Mg^2+^ by grafted plants is also explained by uptake mechanisms that exclude monovalent (Na^+^) ions relative to divalent (Ca^2+^ and Mg^2+^) ions. The increased Mg^2+^ uptake observed in the SO_4_^2−^Cl^−^ water treatments is explained by the greater Mg^2+^ content in those irrigation waters relative to the Cl^−^ waters. Although high concentrations of K^+^, considered luxuriant uptake, can lead to Ca^2+^ and Mg^2+^ deficiency^31^, in our experiment high K^+^ in the Maxifort grafted plants was also associated with increased uptake of Ca^2+^ and Mg^2+^ and decreased uptake of Na^+^. Our leaf K values for nongrafted cv Angela plants was in agreement with results observed by Unlukara, *et al*.^38^ for nongrafted eggplant cv Kemer.

The increased salt tolerance of eggplant grafted to tomato cv. Maxifort is attributed primarily to decreased Na^+^ leaf ion concentration and increased K^+^ and Ca^2+^ leaf concentrations. The lack of a difference in yield between plants irrigated with Cl^−^ as opposed to SO_4_^2−^Cl^−^ waters and the lack of a correlation between leaf Cl^−^ and fruit yield strongly indicates that the improved salt tolerance of eggplant grafted plants is not related to Cl^−^ ion toxicity. Future improvements in eggplant salt tolerance can be focused on improved Na^+^ ion exclusion mechanism or genes related to that process.
